# The novel long noncoding RNA u50535 promotes colorectal cancer growth and metastasis by regulating CCL20

**DOI:** 10.1038/s41419-018-0771-y

**Published:** 2018-07-03

**Authors:** Xihu Yu, Zixu Yuan, Zuli Yang, Daici Chen, Taewan Kim, Yanmei Cui, Qianxin Luo, Zhihang Liu, Zihuan Yang, Xinjuan Fan, DianKe Chen, Lei Wang

**Affiliations:** 1 Guangdong Institute of Gastroenterology, Guangzhou, China; 2 Guangdong Provincial Key Laboratory of Colorectal and Pelvic Floor Diseases, The Sixth Affiliated Hospital, Sun Yat-sen University, Guangzhou, China; 3 Department of Gastrointestinal Surgery, The Sixth Affiliated Hospital, Sun Yat-sen University, Guangzhou, China; 4 The Ohio State University Comprehensive Cancer Center, Columbus, OH USA; 5 Department of Pathology, The Sixth Affiliated Hospital, Sun Yat-sen University, Guangzhou, China; 6 Department of Medical Oncology, The Sixth Affiliated Hospital, Sun Yat-sen University, Guangzhou, China

## Abstract

Long noncoding RNAs (lncRNAs) have been emerging as master regulators of tumor growth and metastasis, but the functions and underlying mechanisms of lncRNAs in colorectal cancer (CRC) still need to be clarified. Here, we found a novel lncRNA u50535, which was greatly overexpressed in CRC tissues and was associated with poor prognosis in CRC patients. Function studies showed that u50535 was an oncogene in CRC both in vitro and in vivo. In mechanism, through RNA sequencing and rescue assay, we found that u50535 activates CCL20 signaling to promote cell proliferation and migration in CRC. Taken together, these findings suggest that u50535 can promote CRC growth and metastasis and may serve as a potential biomarker in CRC.

## Introduction

Colorectal cancer (CRC) is one of the most common malignancies worldwide and ranks as the third cancer of incidence and mortality rate in United States in 2018 and the fifth in China in 2015^1–3^. CRC is a multi-step disease involving a progressive accumulation of genetic alterations and microenvironment interactions^4^. Although numerous genetic alterations and mechanisms have been revealed in the progression of CRC, such as EGFR pathway and WNT/β-Catenin pathway, the molecular mechanisms still remain largely unknown and prognosis of patients is still not yet satisfactory^5, 6^. Therefore, a further understanding of the mechanisms and identifying novel biomarkers for CRC are still on demand.

High-throughput sequencing of whole genomes and transcriptomes has revealed that less than 2% of the genome encodes proteins and at least 75% are actively transcribed into noncoding RNA (ncRNA)^7, 8^. Thus, previous studies focusing on only 2% of protein-coding genes are insufficient to clarify the cancer mechanisms and develop useful biomarkers for clinical practice.^9, 10^. Based on the size of ncRNA, they can be classified into small ncRNA (<200 bps, e.g., miRNA, siRNA and piRNA) and long noncoding RNA (lncRNA) (>200 bps, e.g., lincRNA, macroRNA)^11^. Compared with small ncRNA such as miRNA, lncRNAs possessing more than 200 nucleotides have more complicated and various mechanisms suggesting variety of their roles in tumor carcinogenesis and development^11, 12^. Indeed, lncRNAs have been emerging as master regulators of tumor progression and metastasis, which involves in the regulations of chromatin organization, transcriptional and post-transcriptional levels^13–16^. Moreover, subcellular location of lncRNAs is of great importance to determine their functions^8^. For instance, nucleus lncRNAs tend to be implicated in transcriptional and epigenetic regulations, such as chromatin regulation in cis or trans. LncRNA CDKN2B-AS1 (ANRIL) can cis-regulate CDKN2B on the same chromosome locus by binding to H3K27me/CBX7/PRC1 complex and induce cell proliferation^17^. LncRNA HOTAIR can trans-regulate HOXD expression on different chromosome by interacting with H3K27me3/LSD1/PRC2 complex to promote cancer metastasis^18^. On the other hand, cytoplasmic lncRNAs tend to act at the post-transcriptional level such as splicing regulation and microRNA sponges^19–21^. LncRNA PTENP1 can interact with microRNAs which bind to PTEN mRNA, resulting in expressional activation of the tumor suppressor PTEN^22^. Therefore, lncRNAs are believed to be potential cancer biomarkers and tumor therapeutic targets.

Recently the inflammatory microenvironment has been found to be involved in CRC tumorigenesis^23, 24^. CCL20 is one of the main chemokines in tumor microenvironment^23, 25, 26^. CCL20 secreted by tumor cells can target not only immune cells but also tumor cells themselves, which have been shown to regulate tumor cell proliferation, cancer invasion and metastasis by stimulating CCR6-NFkB signaling and PI3K/AKT-ERK signaling^24, 27, 28^. This reveals new strategies to treat CRC by targeting the inflammatory pathway. In the present study, we found that higher expression of u50535 is correlated with poor prognosis of CRC patients. Our finding shows that u50535 serves as an oncogene in CRC progression and metastasis by enhancing cell proliferation, migration and invasion. We also reveal that u50535 can regulate CCL20 expression by regulating its promoter activity and affect CCL20/CCR6/ERK signaling, leading to CRC tumorigenesis.

## Results

### LncRNA-u50535 expression is frequently increased and is associated with poor prognosis of CRC

**Fig. 1:**
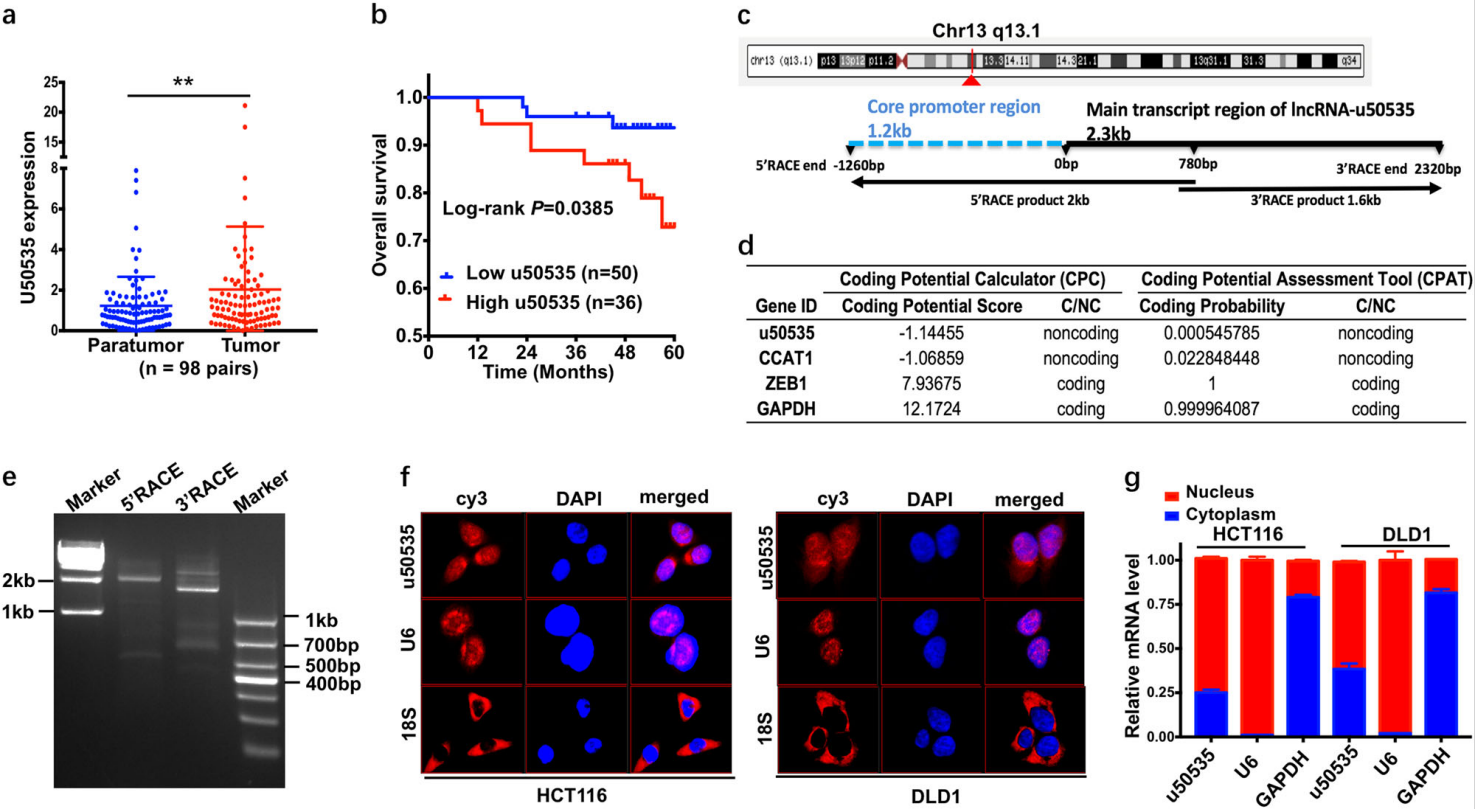
Increased novel lncRNA-u50535, which locates mostly in nucleus and partly in cytoplasm, was associated with poor prognosis. **a** u50535 expression was measured in 98 paired CRC tumor (T) and paratumor normal tissues (P). The u50535 expression in T was significantly higher than P (*P* value = 0.0076). **b** Higher expression of u50535 obtained worse overall survival than lower expression (*n* = 86). **c**, **e** RACE experiment showed the 5′ and 3′ RACE product of u50535 and the sequencing results were consistent with UCSC database. In addition, the longer sequence in 5′RACE was the promoter region and the details were shown in Fig.S2. **d** u50535 was predicted to be non-coding RNA in CPC and CPAT tools. **f** FISH analysis showed the sub-cellular location of u50535 in HCT116 and DLD1. U6, nucleus location positive control. 18S, cytoplasm control. The subcellular location of u50535 is mostly in nucleus and partly in cytoplasm. **g** u50535 expression in the nucleus and cytoplasm fractions by qRT-PCR were consistent with FISH results. Data from three independent experiments were expressed as mean ± SD

We previously have published the lncRNA expression profiles in six pairs of CRC and the adjacent normal tissues by lncRNA microarray, and lncRNA-u50535 ranked as one of the top ten remarkably upregulated genes with 6.04-fold increase^29^. To investigate the role of the novel lncRNA-u50535 in the clinical progression of CRC, we firstly examined mRNA expression of u50535 in additional 98 paired CRC tumor and paratumor normal tissues. As shown in Fig. 1a, the u50535 expression was significantly increased in tumor tissues compared with paratumor normal tissues (*P* = 0.0076).

In addition, based on the receiver operating characteristic (ROC) curve analysis, the patients were further divided into high and low expression groups. In the total of 98 patients, 12 were excluded for the clinical parameters analysis, including three who died of non-cancerous causes, and nine who had pre-operative distant metastasis and did not undergo radical metastatic site resection. Chi-square tests revealed that high u50535 expression level is strongly correlated with the lymph node metastasis (*P* = 0.025), and TNM stage (*P* = 0.037) (Table 1).

**Table. 1:** The correlations between lncRNA-u50535 and clinicopathological characteristics in 86 CRC cases

Characteristics	lncRNA-u50535		
	Low (*n* = 50)	High (*n* = 36)	*P* value
Age			0.799
<60	25	17	
≥60	25	19	
Gender			0.424
Female	14	13	
Male	36	23	
Location			0.982
Colon	36	26	
Rectum	14	10	
Colorectal cancer			0.095
Right side	18	7	
Left side	32	29	
Depth of invasion			0.579
T1/T2	5	5	
T3/T4	45	31	
Lymph node metastasis			0.025^*^
N0	38	19	
N1–2	12	17	
Distant metastasis			0.624
Absent	46	32	
Present	4	4	
TNM stage			0.037^*^
I/II	36	18	
III/IV	14	18	
Histological grade			0.271
Well/moderately	40	32	
Poorly	10	4	
Vein/nerve invasion			0.281
No	41	26	
Yes	9	10	
Tumor size			0.690
≤5 cm	27	21	
>5 cm	23	15	

^*^P < 0.05. Analysis with Chi-square test in SPSS 22

As for survival and prognosis relationship, patients with high u50535 level had significantly poor 5-year over-all survival (OS) than those with low u50535 level (*P* = 0.0385) (Fig.1b). Univariate survival analysis identified three prognostic indicators: lymph node metastasis, TNM stage and u50535 expression. Multivariate analysis further revealed that u50535 expression could be an independent predictor for CRC prognosis (*P* = 0.041) (Table.2). These observations suggest that increased u50535 expression is closely associated with poor prognosis of CRC and that it could be a novel biomarker for the diagnosis and prognosis of CRC.

**Table. 2:** Univariate and multivariate analysis of clinicopathological parameters for overall survival

Factors	Univariate analysis		Multivariate analysis	
	RR	*P* value	RR	*P* value
Age (<60/≥60)	1.930 (0.577–6.451)	0.286		
Gender (female/male)	1.326 (0.351–5.001)	0.677		
Location (colon/rectum)	1.086 (0.316–3.729)	0.896		
CRC (right/left side)	1.080 (0.286–4.084)	0.909		
T stage (T1 + T2/T3 + T4)	23.948 (0.005–114)	0.462		
N stage (N0/N1 + N2)	4.011 (1.170–13.749)	0.027^*^		
Distant metastasis (absent/present)	2.792 (0.598–13.031)	0.191	2.745 (0.584–12.900)	0.20
TNM stage (I + II/III + IV)	3.518 (1.025–12.083)	0.046^*^		
Histological grade (well + moderate/ poor)	1.820 (0.482–6.874)	0.377		
Vein/nerve invasion (no/yes)	1.209 (0.321–4.559)	0.779		
Tumor size (≤5/>5 cm)	0.835 (0.262–2.659)	0.760		
lncRNA-u50535 expression (low/high)	4.029 (1.067–15.205)	0.040^*^	4.008 (1.061–15.141)	0.041^*^

*RR* relative risk, ^*^ *P* < 0.05. Analysis with univariate and multivariate Cox regression in SPSS 22

### The u50535 is a novel long noncoding RNA mostly located in nucleus

To identify the actual sequence of this novel lncRNA-u50535 in CRC, we conducted 5′ and 3′RACE in CRC-derived cell lines and the RACE products were determined by direct sequencing (Fig. 1c, e). The sequencing results of u50535, which located in chromatin 13q13.1, were consistent with UCSC database. In addition, 3′RACE sequencing indicated that u50535 had a poly(A) tail. Besides, the longer sequence on 5′RACE is the promoter region of u50535 and the detailed explanation is shown in supplementary Fig.S2.

Furthermore, both coding potential calculator (CPC) and coding potential assessment tool (CPAT) analysis revealed that u50535 has less potential to encode protein (Fig. 1d). Both FISH and subcellular fractionation location assays indicate that u50535 is mostly located in the nucleus in CRC-derived cell lines such as HCT116 and DLD1 cells (Fig. 1f, g). This implies the main function of u50535 at the transcriptional level.

### LncRNA-u50535 enhances CRC cell proliferation in vitro

**Fig. 2:**
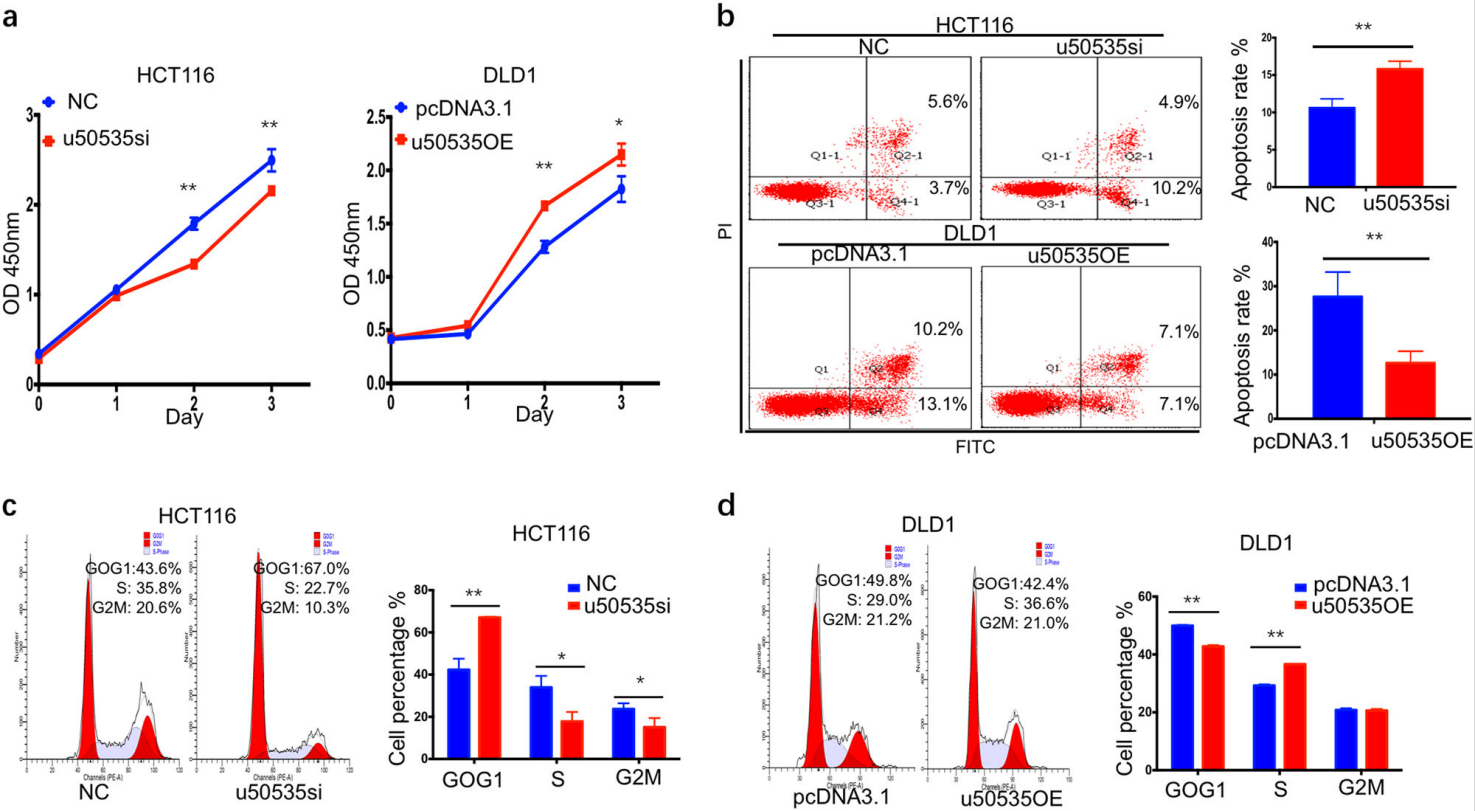
u50535 enhances CRC cell proliferation, inhibits apoptosis and accelerates cell cycle. **a** CCK8 assays revealed that u50535 silence inhibited HCT116 cell proliferation, while u50535 overexpression promoted DLD1 cell proliferation. **b** Apoptosis assays by flow cytometry indicated that u50535 silence increased apoptosis rate in HCT116, while u50535 overexpression decreased apoptosis rate in DLD1. **c, d** Cells in G0/G1 phase were mainly non-proliferation cells. u50535 silence increased the percentage of HCT116 cells in G0/G1 phase, while the overexpression of u50535 inhibited the percentage of DLD1 cells in G0/G1 phase. Data from three independent experiments were expressed as mean ± SD. * *P* < 0.05; ** *P* < 0.01

To investigate the impact of u50535 in CRC cells, we examined the u50535 expression in four CRC cell lines and one paratumor normal tissue as a relative control (supplementary Fig. S1a). The cell lines such as HCT116 and HCT8 harboring higher u50535 expression were subjected to knockdown experiments through siRNA transfection, and overexpression experiments of u50535 were performed in the cell lines (HCT15 and DLD1) with relatively low u50535 expression through pcDNA3.1-u50535 transfection (supplementary Fig. S1b, c). Silence of u50535 in CRC cells significantly inhibited cell proliferation, whereas overexpression of u50535 accelerated cell proliferation by CCK8 assay (Fig. 2a, supplementary Fig. S3a).

We next investigated the role of u50535 on cell apoptosis and cell cycle. Silence of u50535 in CRC cells can induce cell apoptosis and also increase the proportion of cell population in G0/G1 phase in HCT116 and HCT8, which give rise to the inhibition of cell proliferation. Consistently, overexpression of u50535 in DLD1 and HCT15 decreased cell apoptosis and the proportion of cell population in G0/G1 phase (Fig. 2b-d, supplementary Fig. S3b-d). Taken together, these results indicate that u50535 enhances CRC cell proliferation through the inhibition of cell apoptosis and cell cycle G0/G1 arrest.

### LncRNA-u50535 promotes CRC cell migration, invasion and morphology change in vitro

**Fig. 3:**
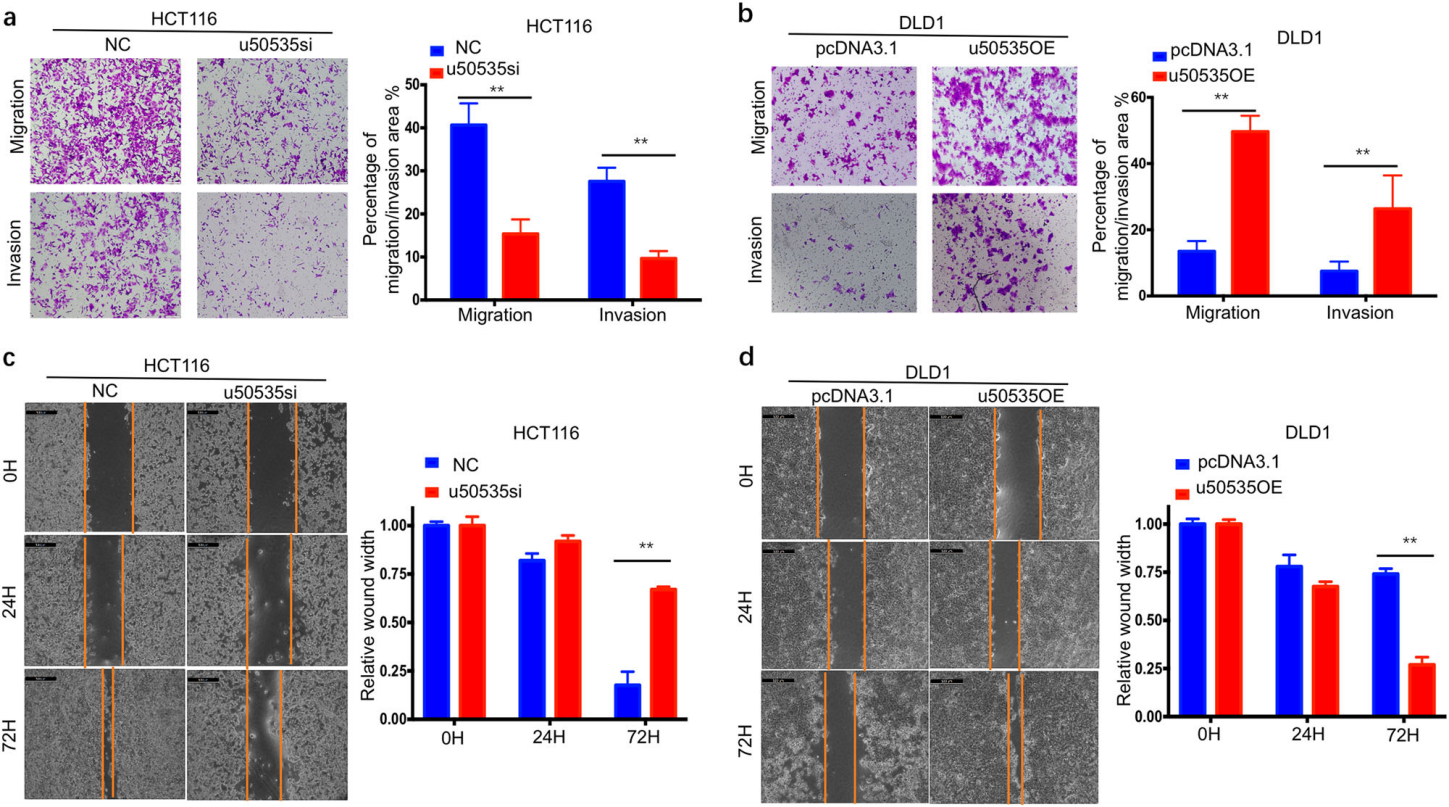
u50535 promotes CRC cell invasion and migration. **a**, **b** Transwell assays revealed that u50535 knockdown repressed both migration and invasion ability in HCT116, while opposite results were found in u50535 overexpressed DLD1. **c**, **d** Wound healing test showed that u50535 knockdown would significantly slow down the ability of wound healing in HCT116, while its overexpression greatly promoted the wound healing in DLD1. Data from three independent experiments and expressed as mean ± SD. * *P* < 0.05; ** *P* < 0.01

To determine whether u50535 regulates cell migration and invasion, we carried out the transwell assays. Silence of u50535 significantly repressed both cell migration and invasion in HCT116 and HCT8 cells (Fig. 3a, supplementary Fig. S4a), whereas opposite results were investigated by the overexpression of u50535 in DLD1 and HCT15 cells (Fig. 3b, supplementary Fig. S4b). Consistently, wound healing test showed that u50535 knockdown inhibits the wound healing ability of HCT116 and DLD1, whereas overexpression of u50535 accelerates the wound healing ability of DLD1 (Fig. 3c, d, supplementary Fig. S4c). Additionally, cell morphology has been captured by microscope. Silence of u50535 by siRNA would result in a smoother cell surface, more tightly packed cells, while overexpression of u50535 would lead to an elongated and spindle-like cell surface, more free or loose cells (supplementary Fig. S4e). Stably overexpression of u50535 by lentivirus seemed to be more obvious and showed a rougher cell surface with some pseudopods compared with control cells (supplementary Fig. S4d). Taken together, these data suggest that u50535 promotes CRC cell migration, invasion and morphology change.

### LncRNA-u50535 overexpression increases tumor growth and lung metastasis in vivo

**Fig. 4:**
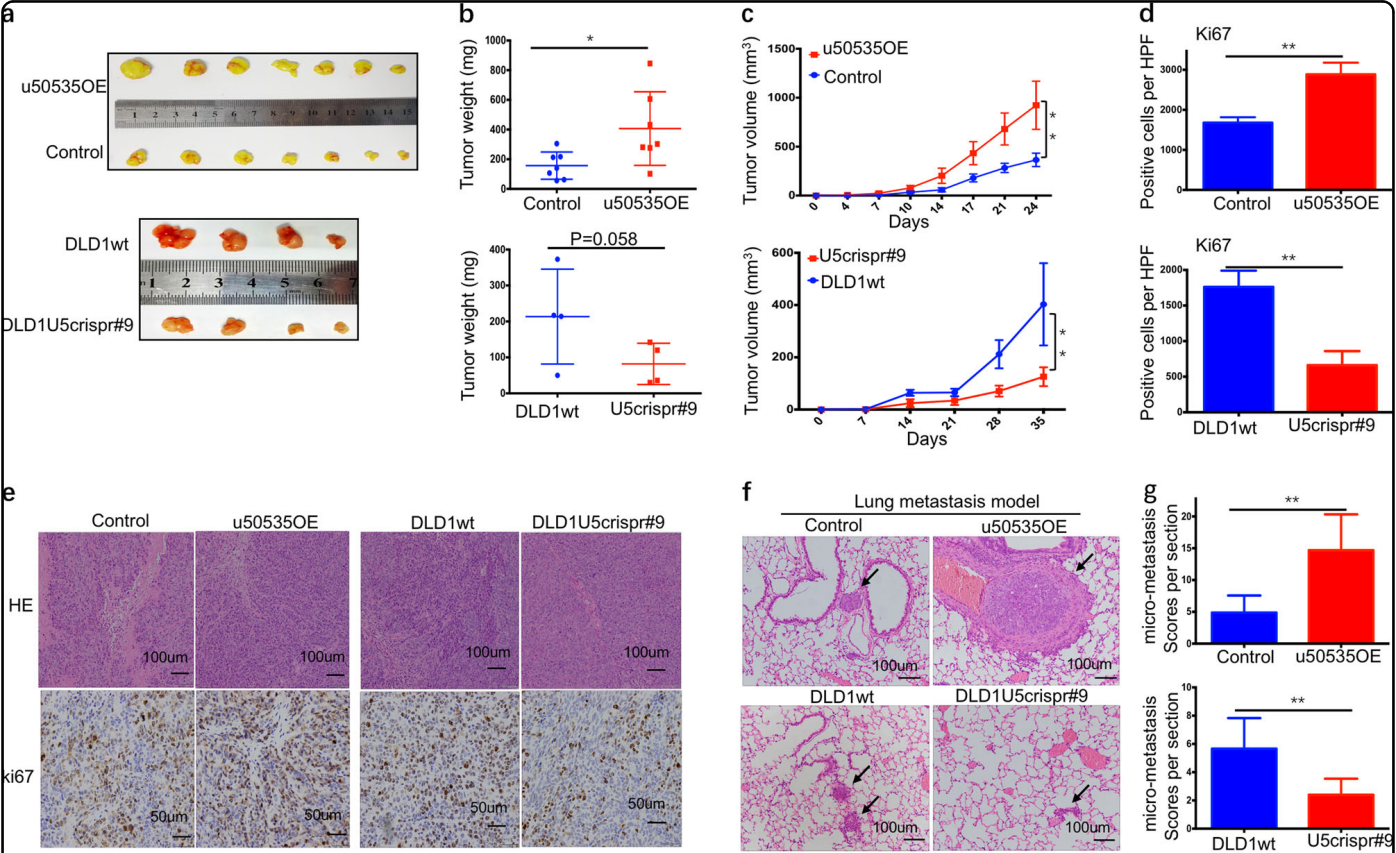
u50535 increases tumor growth and lung metastasis in vivo. **a** The u50535 overexpressed cells (upper panel, *n* = 7) or knockout cells (down panel, *n* = 4) and their respective controls were injected into BALB/c mice. **b**, **c** Tumor weights and tumor volumes were calculated. Tumor weights and volumes were greatly increased in u50535OE group, while decreased in u50535 knockout group. **d**, **e** Representative H&E images of histopathology of xenograft tumor were presented. Ki67 positive cells numbers per 40× high power field (HPF) were calculated. **f**, **g** Representative H&E images of metastasis tumor nodules were presented. Lung micro-metastasis was significantly increased in u50535 overexpression group (*n* = 5), while decreased in u50535 knockout group (*n* = 5). * *P* < 0.05; ** *P* < 0.01

To confirm the effect of u50535 on tumor growth, we constructed the xenograft tumor model by subcutaneous injection of PCDH-control cells and u50535 overexpressed (OE) cells. Compared with control, the u500535OE cells exhibited more rapid rate of tumor growth, including tumor weight and tumor volumes (Fig. 4a-c). Moreover, IHC staining revealed that proliferation marker Ki67 expression is increased in the u50535OE xenograft tumor tissues (Fig. 4d, e). We also constructed the gene knockout xenograft tumor model by using dual-sgRNA CRISPR/Cas9 method. As expected, opposite results were investigated in tumor weight, tumor volumes and Ki67 index after u50535 knockdown (Fig. 4a-e).

To determine the effect of u50535 on tumor metastasis in vivo, we also generated lung metastasis model through tail vein injection of overexpression or knockout cells. For the lung metastasis, the score of lung micro-metastasis was increased in the u50535 overexpression group compared with the control group. On the contrary, u50535 knockout caused a decreased score of lung micro-metastasis (Fig. 4f). Taken together, these data support that u50535 enhances tumor growth and metastasis in CRC.

### The potential downstream signaling of u50535

**Fig. 5:**
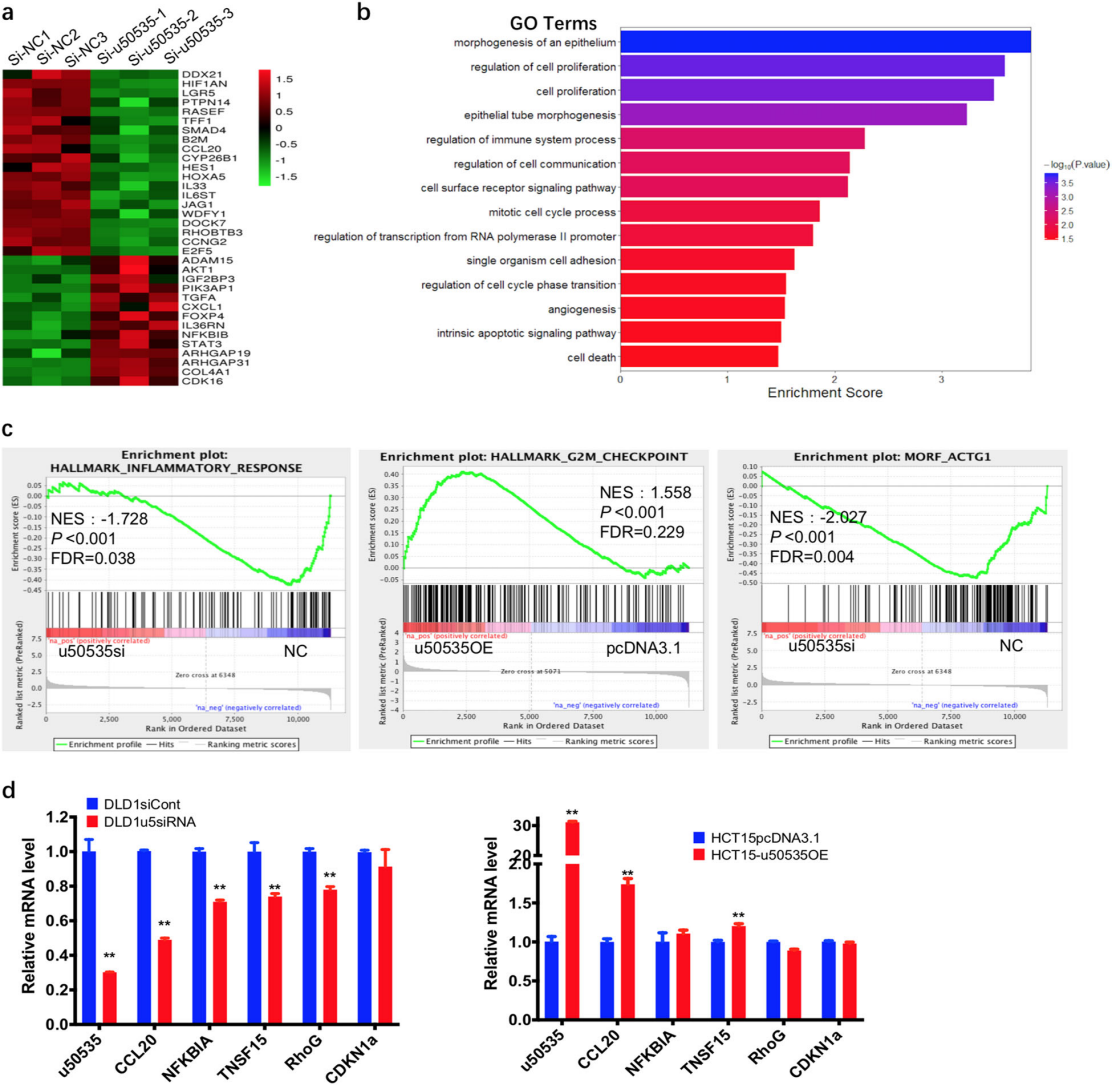
RNAseq reveals potential downstream signaling of u50535. **a** Heat map displays altered expressions of 333 genes (≥1.5-fold change) in si-NC-treated and siRNA-u50535-treated DLD1 cells (details genes in supplementary Table. S2). **b** Gene ontology (GO) analysis of the altered genes were involved in biological processes (BP) function, which include cell proliferation, cell cycle, apoptosis, adhesion, morphology, immune system and so on. **c** GSEA reveals enrichment of u50535 associated genes in “HALLMARK INFLAMMTORY RESPONSE”, “HALLMARK G2M CHECKPOINT”, and “MORF ACTG1”. **d** The altered genes selected from GSEA were confirmed by real-time PCR in knockdown or overexpression of u50535

To investigate the mechanism of u50535 in promoting CRC progression, firstly, we performed the RNAseq assays to evaluate gene expression changes triggered by u50535 inhibition or overexpression. Heat map plots revealed 333 genes (≥1.5-fold change) were dysregulated after u50535 knockdown (Fig. 5a, supplementary TableS2). Then gene ontology (GO) method was performed to analyze related biological processes function of these identified genes (Fig. 5b). Consistent with our observation in vitro and in vivo, GO results also show that the u50535-regulated genes were mostly involved in pathways like cell proliferation, cell cycle, apoptosis, adhesion, morphology, immune system and so on. As GO analysis only included the significant genes, we further confirmed the potential pathway by GSEA preranked tool analysis including all genes. GSEA revealed the enrichment of u50535 was associated with genes in “HALLMARK INFLAMMATORY RESPONSE”, “HALLMARK G2M CHECKPOINT”, and “MORF ACTG1” (Morphology ActinG1) (Fig. 5c). Furthermore, the altered genes were selectively confirmed by qRT-PCR and found similar altered gene expressions to GSEA analysis in u50535 knockdown or overexpression cells (Fig. 5d, supplementary Fig. S5). Among these dysregulated genes, CCL20 expression exhibited the highest fold change and thus attracted our attention.

### LncRNA-u50535 promotes CRC growth and metastasis via activation of CCL20 signaling

**Fig. 6:**
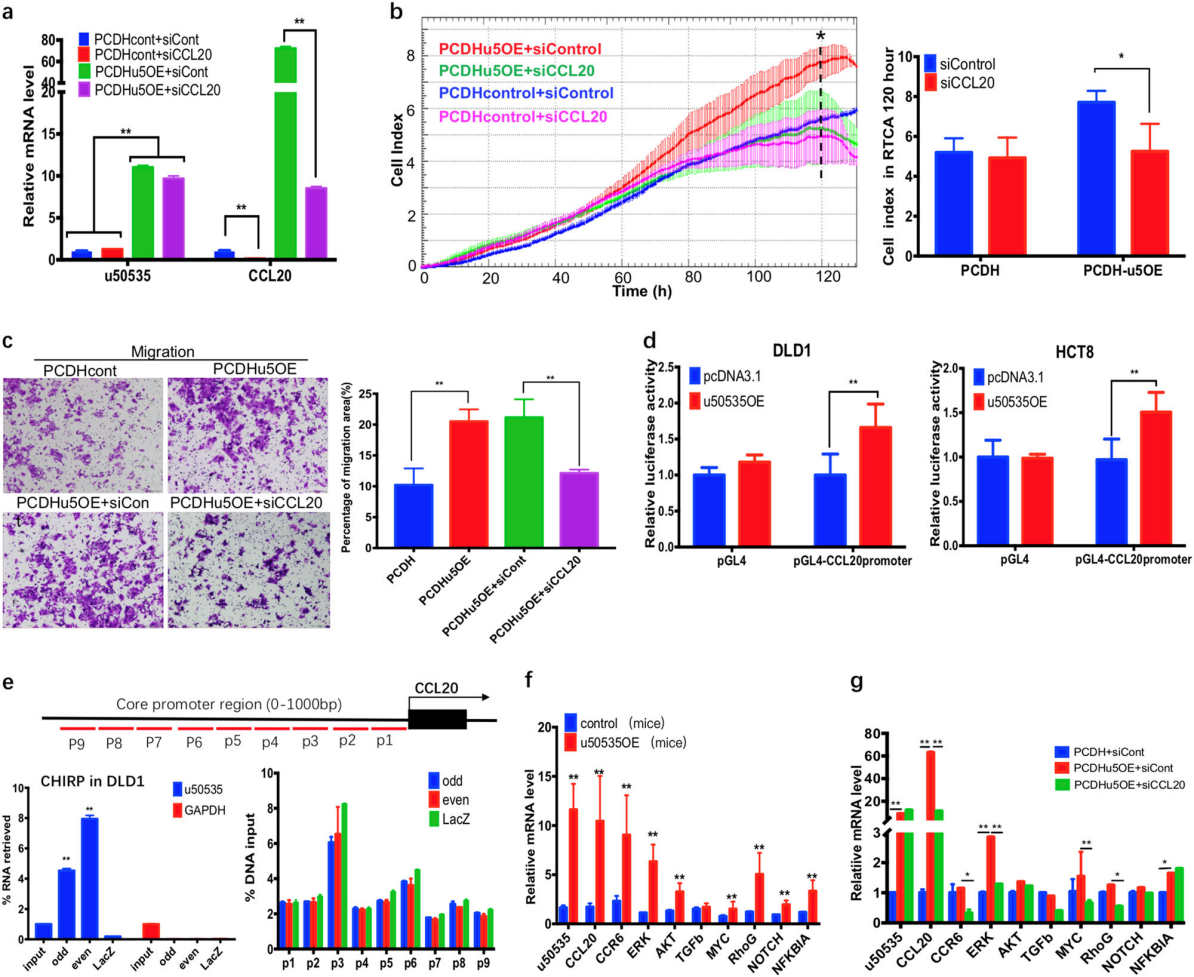
u50535 promotes CRC growth and metastasis via activation of CCL20 signaling. **a** After transfecting siCCL20 to u50535 stable overexpression cells (PCDHu5OE), the expression of u50535 was not affected after down-regulation of CCL20, whereas the expression of CCL20 was significantly decreased. **b** Proliferation assay by RTCA showed that CCL20 knockdown can reverse the proliferation activity induced by u50535 overexpression (PCDHu5OE), especially in 120 h. **c** Transwell assay showed that CCL20 knockdown can inhibit the migration ability caused by u50535 overexpression. **d** Luciferase assay showed that relative luciferase activity of pGL4-CCL20-promoter was significantly increased by u50535 overexpression in DLD1 and HCT8, supporting that u50535 could positively regulate CCL20 transcription directly or indirectly. **e** CHIRP assay revealed that u50535 was significantly retrieved by u50535 antisense odd/even probes compared with negative control LacZ in DLD1 (left), while chromatin/DNA of CCL20 core promoter region was not significantly enriched (right), indicating an indirect regulation. **f** The mRNA expression of CCL20 and related genes were testified in xenograft tumor from control/u50535OE mice model. **g** u50535 overexpression in DLD1 cells can significantly promote CCL20, ERK, NFKBIA genes expression, while adding siRNA-CCL20 greatly inhibits the expression of CCL20, CCR6 and ERK. Data from three independent experiments were expressed as mean ± SD. * *P* < 0.05; ** *P* < 0.01

To reveal the mechanism of u50535 in regulating CCL20 expression, we firstly evaluated the mRNA expression relationships between u50535 and CCL20 after transfecting siRNA-CCL20 to u50535 overexpression cells (Fig. 6a). The results revealed that u50535 can upregulate CCL20, but CCL20 expression cannot reversely affect u50535 expression. These results indicated that u50535 might be the upstream regulator of CCL20. Then, rescue assays by proliferation assay (real-time cellular analysis, RTCA) and transwell assays revealed that overexpression of u50535, which promotes tumor cell growth and migration, could be reversed after CCL20 downregulation (Fig. 6b, c). Thus, u50535 can promote tumor cell proliferation and migration by regulating CCL20 expression.

Previous studies showed that nucleus lncRNA can regulate gene expression by serving as a promoter regulator^30, 31^. To investigate whether u50535 affects the promoter activity of CCL20, we performed luciferase assay. Results revealed that relative luciferase activity of pGL4-CCL20-promoter was significantly increased by u50535 overexpression, supporting that u50535 could positively regulate CCL20 transcription directly or indirectly (Fig. 6d). Next, we conducted chromatin isolation by RNA purification (CHIRP) assay to explore whether u50535 directly binds and regulates CCL20 promoter region. The results revealed that lncRNA u50535 was significantly retrieved and enriched by u50535 antisense odd/even probes compared with negative control LacZ in DLD1 cells and u50535OE cells. But the chromatin enrichment on CCL20 promoter region was not significantly upregulated (Fig. 6e, supplementary Fig. S6), which indicated that u50535 may indirectly regulate CCL20 promoter activity.

Previous studies reported that CCL20 could promote tumor cell proliferation, invasion and metastasis by stimulating CCR6/NFkb signaling and PI3K/AKT-ERK signaling^27, 28^. To further explore the relationship between u50535 and the potential related signaling of CCL20 both in vivo and in vitro. In vivo, we testified gene expression in xenograft tumor from control/u50535OE mice model and found that the u50535 overexpression can upregulate CCL20 and its following signal molecules such as CCR6, ERK, AKT, NFKBIA and so on (Fig. 6f). In vitro, u50535 overexpression in DLD1 cells can significantly promote CCL20, ERK, NFKBIA genes expression, while adding siRNA-CCL20 greatly inhibits the expression of CCL20, CCR6, and ERK (Fig. 6g). These results showed that u50535 can regulate CCL20 expression and affect CCL20/CCR6/ERK signaling. Taken together, these findings indicate that u50535 may promote CRC growth and metastasis via activation of CCL20 signaling.

## Discussion

Colorectal cancer is a multi-gene and multi-factor contributory disease^3^. In the past, most studies focused on coding proteins that accounted for less than 2% of the human genome, while the remaining 75% of non-coding RNAs that are transcriptionally active are ignored^9^. This greatly restricts the deep understanding of CRC and the discovery of new potential targets for diagnosis and treatment. In recent years, more and more research evidences showed that lncRNAs are closely related to the carcinogenesis and development of CRC and are expected to become new molecular markers and therapeutic targets of CRC^16^. In this study, we reported an upregulated novel lncRNA-u50535 in CRC based on our previously published lncRNA microarray^29^. The high expression of u50535 is significantly correlated with lymph node metastasis and advanced TNM stage, as well as poor survival of patients. These results indicated that u50535 might be an independent diagnostic and prognostic marker for CRC patients.

Based on UCSC and NCBI database, we find that lncRNA-u50535 is located in human chromosome 13q13.1 and is an intronic lncRNA of host gene N4BP2L2 in human BRAC2 region. Although there is no publication about the relationship between N4BP2L2 and tumor, many studies have showed that BRAC2 is an oncogene that is strongly associated with development of breast cancer and prostate cancer^32, 33^. Thus, we hypothesized that lncRNA-u50535 in the inter region of BRAC2 may also play important roles in development of cancer. In the present study, we found that overexpression of u50535 could promote cell proliferation, invasion and migration in CRC both in vitro and in vivo. This indicates that u50535 acts as an oncogene in CRC development, which is consistent with our previous hypothesis. In mechanism, in order to explore u50535-regulated genes and pathways in a full-scale method, we performed the RNAseq assays and conducted GSEA analysis, which revealed that u50535 can influence CRC carcinogenesis through affecting the pathways of cell proliferation, cell cycle, inflammation, cell adhesion and cell morphology. Further qRT-PCR validation of the potential genes from GSEA analysis suggested that CCL20 may be a potential target of u50535.

CCL20 is one kind of chemokines and is expressed in a variety of human tissues such as a variety of immune cells, normal tissues of colon, stomach and so on^24, 25^. And its chemokine receptor is CCR6. Frick et al. reported that CRC cells express both CCL20 and CCR6 in a non-polarized manner, providing a basis for efficient paracrine and autocrine loops^25^. Moreover, Brand et al. and Cheng et al. demonstrates that CCL20 overexpression can promote CRC via CCR6 which mediates PI3K/ERK/AKT signaling pathway^27, 28^. Jin et al. shows that CCL20 promotes glioblastoma by stimulating CCR6/NFkb signaling^34^. In our study, we found that u50535 can positively regulate the expression of CCL20. After knockdown of CCL20 in u50535 overexpressed cells, we found that CCL20 can abrogate the effects of proliferation and migration that are induced by u50535 in CRC cells. These results indicate u50535 can regulate CCL20 both in expression and function. Furthermore, u50535 is mainly located in nucleus. Huang et al. indicated that nucleus lncRNA lncAKHE can enrich on NOTCH2 promoter and activate NOTCH2 expression and signaling^31^. Thus, we have a similar hypothesis that nucleus lncRNA u50535 may also regulate CCL20 expression by regulating its promoter activity. The luciferase assays, which is commonly used as promoter binding assay, have indicated that u50535 may positively regulate CCL20 promoter activity directly or indirectly. However, the CHIRP assay showed that u50535 was not directly enriched on CCL20 promoter region. u50535 may regulate CCL20 in an indirect manner. For example, u50535 may activate another molecule which in turn induces the expression of CCL20. Also, increasing evidences indicated that lncRNA can act as a scaffold to recruit proteins for regulating gene expression^30^. So the recruited proteins may be links between u50535 and CCL20. But further investigations are needed.

However, there are some limitations in this study. Firstly, no CCL20 inhibitor or stimulator was added to construct the animal model, which will limit the ability to capture the effect of CCL20 on u50535′s function. However, we further explored the relationship between u50535 and the potential related signaling of CCL20 both in vivo and in vitro. We found that u50535 can regulate CCL20, CCR6, ERK, AKT and NFKBIA expression, which is consistent with the previous studies that report that CCL20 triggers CCR6/PI3K/ERK-AKT signaling or CCR6/NFkb signaling^28, 34^. Therefore, CCL20 induced by u50535 may contribute to CRC tumorigenesis. By the way, although the BALB/c nude mice we used in the study are immunodeficient, CCL20 can still promote CRC tumorigenesis via non-immune pathways such as CCR6/PI3K/ERK-AKT signaling. Moreover, CRC cells can express both CCL20 and CCR6 and provide an efficient paracrine and autocrine signaling loops without the help of immune cells.

Secondly, the mechanism of overexpression of u50535 is not investigated in this study. Recent studies have reported that the cell-type-specific expression of lncRNA may be regulated by epigenetic modifications such as histone modifications in a similar way to protein-coding genes^35^. For instance, Zhang et al. indicated that lncRNA CCAT1 was activated by H3K27 acetylation in esophageal carcinoma^36^. Ding et al. found that lncRNA GHET1 was also activated by H3K27Ac in liver cancer^37^. We explored the UCSC database, and found that H3K4me3 and H3K27Ac are highly enriched at u50535 nearby promoter region (supplementary Fig. S2a). Thus, we hypothesize that u50535 expression may be also activated by histone modification such as H3K4me3 and H3K27Ac. This needs further verification.

In conclusion, our study indicates that u50535, which is frequently increased in CRC tissues and associated with poor prognosis, is a novel lncRNA that promotes tumor progression and metastasis. In mechanism, u50535 promotes CRC tumorigenesis partly by regulating CCL20 signaling pathway. Taken together, these findings suggest that u50535 is an oncogene in CRC and may serve as a potentially prognostic and therapeutic target for CRC patients.

## Materials and methods

### Patients and sample collection

A total of 98 pairs tumor and paratumor tissues (2 cm away from the tumor border) from CRC patients analyzed in this study were obtained from the Tissue Bank, Sixth Affiliated Hospital of Sun Yat-sen University (SYSU), China, approved by Human Medical Ethics Committee of SYSU and attached with informed consents from all patients^38^. Clinical–pathological parameters and follow-up information were collected from the Follow-up database of Sixth Affiliated Hospital of SYSU and confirmed by checking the original medical records manually.

### Cell culture

Human CRC cell lines (HCT116, HCT8, DLD1, HCT15), purchased from the American Type Culture Collection (ATCC) are all cultured in RPMI-1640 medium (Gibco, USA) with 10% FBS (Gibco, USA) and 1% penicillin/streptomycin (Invitrogen, Carlsbad, CA, USA) in a humidified incubator at 37 °C with 5% CO_2_.

### RNA extraction, qRT-PCR and western blot assays

Total RNA was extracted from tissues or cells with TRIzol reagent (Invitrogen) according to manufacturer’s instructions and then was reversely transcribed as cDNA with ReverTra Ace qPCR RT kit (Toyobo, Japan). Quantitative real-time PCR (qRT-PCR) analysis were performed with FastStart Essential DNA Green Master (Rhoche, Germany) in LightCycler 96 Instrument (Rhoche) with the following conditions: 95 °C for 10 min and 40 cycles of 95 °C 15 s, 60 °C 60 s. Gene expression was normalized as 2^-∆∆CT^ and CT value was compared to GAPDH. The primer sequences were showed in supplemental data (Table S1).

Protein was extracted by T-PER tissue protein extraction reagent (Thermo, Rockford, IL, USA) with protease inhibitor cocktail set III and phosphatase inhibitor cocktail set II (Millipore, Germany) according to manufacturer’s instructions. Primary antibodies included anti-ZEB1(CST, #3396), anti-N-Cadherin (CST, #13116), anti-MMP9 (abcam, ab137867), anti-pAKT-S473 (CST, #9271), anti-pERK (CST, #4370), and anti-GAPDH (Proteintech Group, #10494-1-AP).

### Rapid amplification of cDNA ends (RACE)

RACE was performed with SMART RACE cDNA Amplification Kit (Clontech, USA) according to manufacturer’s instructions. Total RNAs from HCT116 and RKO were used. RACE of the overlap region in the middle of u50535 was the 120 bp PCR product, which has been confirmed by direct sequencing. Thus, we designed the RACE primers based on this known region. Primers for RACE were presented in supplemental data Table S1.

### RNA fluorescent in situ hybridization (FISH)

RNA FISH assay was performed with Ribo Fluorescent in Situ Hybridization Kit (Ribo, China) according to manufacturer’s instructions. The lncRNA probe mix of u50535 and positive nucleus control probe U6 and positive cytoplasm control probe 18S were designed and purchased from Ribo company. The FISH results were captured by confocal instrument (Leica TCS-SP8).

### Subcellular fraction location

The separation of nucleus and cytoplasm fractions was performed with NE-PER nuclear and cytoplasmic extraction reagents (Thermo, #78833, USA) according to manufacturer’s instructions. Then we testified the mRNA expression of u50535, U6, GAPDH in nucleus and cytoplasm by qRT-PCR.

### **s**iRNA and overexpression transfection

The siRNAs of u50535 and CCL20 and scrambled negative control siRNA (si-NC) were purchased from Ribo company. Sequence of siRNAs were listed in supplementary data. The siRNAs were transfected into CRC cells using Lipofectamine RNAiMAX (Invitrogen) according to manufacturer’s instructions.

The overexpression of u50535 was constructed by inserting u50535 sequence from RACE assay into pcDNA3.1+ plasmid at multiple cloning site with KpnI and XhoI restriction enzymes (New England Biolabs, USA) and Ligation high kit (TOYOBO, LGK-100, Japan). The plasmids were transfected into CRC cells with Lipofectamine 3000 (Invitrogen) according to manufacturer’s instructions.

### Vector construction, lentiviral construction, CRISPR/cas9 and infection

The stable overexpression of u50535 plasmid was constructed by ligating u50535 sequence to PCDH-GFP vector with In-Fusion HD Cloning Kit (Clontech, cat# 011614) according to manufacturer’s instructions. Then PCDH-u50535OE were co-transfected with pCMV-∆8.91 and pCMV-VSVG into 293T cells by using Lipofectamine 3000 to generate viral supernatants. After the concentration of lentivirus by cold high-speed centrifuge, the targeted CRC cells were infected by incubating with lentivirus for 48 h. We selected the overexpression clones with GFP green lights by qRT-PCR confirmation.

The stable knockout of u50535 was established by CRISPR/Cas9 method according to Zhang Lab GeCKO website protocol. We designed the dual sgRNA based on PrecisionX Mutiplex gRNA Cloning Kit (SBI, # CAS9-GRNT-Kit). The sgRNA primers were listed in supplementary data (Table S1). LentiCRISPRv2 vector was ligated to dual sgRNA. The lentivirus was constructed and incubated with CRC cells as above. After puromycin screening, we selected the knockout clones by qRT-PCR confirmation.

### Cell proliferation assay: CCK8 and RTCA

Cell proliferation was performed with Cell Counting Kit-8 (CCK8) (Dojindo Lab, Japan) according to manufacturer’s instructions. 6000 cells/well were seeded into 96-well plate and the proliferation index at 450 nm absorbance was measured every day by Thermo Scientific Varioskan Flash machine after incubating with 10 µl CCK8 solution for 2 h at 37 °C.

Cell proliferation was also performed with real-time cellular analysis (RTCA) assay by xCELLigence DP device (ACEA Biosciences, USA). 6000 cells/well were seeded into E-plates and automatically monitored and recorded every 15 min by the RTCA device.

### Cell apoptosis and cell cycle assays

Cell apoptosis was performed with Annexin V/PI Apoptosis Kit (Multi Sciences, China) according to manufacturer’s instructions. Cell cycle assays was performed with Cell Cycle Staining Solution (Multi Sciences) according to manufacturer’s instructions. The flow cytometry results were measured by BD FACS Calibur device and analyzed with ModFit 3.0 software. Each assay was repeated three times.

### Cell migration and invasion assays

Transwell assay was performed with Cell Culture Insert of 24 well 8.0 µm pore size (Falcon, USA) with or without Matrigel (BD Biosciences, USA). 4 × 10^4^ cells were placed into the upper chamber in 0.2 ml of serum-free RPMI-1640, whereas RPMI-1640 with 10% FBS was placed in the lower chamber as a chemoattractant. Migration and invasion cells in the other side of membrane were fixed and stained with crystal violet for 5 min after 24–48 h culture. Each assay was repeated three times.

As for wound healing assay, 1 × 10^6^ cells were seeded into 12-well plate and wounds were made by a tip of 200 µl pipette. The size of wound was captured every day by microscope and measured by Image J software. Each assay was repeated three times.

### Luciferase reporter assay

Before luciferase reporter assay, pGL4-CCL20-promoter plasmid with promoter region of CCL20 was constructed and then was co-transfected into CRC cells with pRL-TK plasmid and pcDNA3.1-u505535 overexpressed plasmid by Lipofectamine 3000. After incubating for 24–48 h, the luciferase activity was measured by Dual Luciferase Reporter Assay System (Promega, E1910, USA) according to manufacturer’s instructions.

As for the luciferase assay of miR-200 interaction with ZEB1, the 3′-UTR regions of ZEB1 was cloned into psi-CHECK2 vector (Promega) and the miR200 mimic was purchased from Ribo company. The site mutation of pcDNA3.1-u50535 in the region 1706–1712 was performed with KOD-Plus-Mutagenesis Kit (TOYOBO, SMK-101) according to manufacturer’s instructions. psi-CHECK2 was co-transfected into CRC cells with pcDNA3.1-u50535 and miR-200 mimic by Lipofectamine 3000. The luciferase activity was measured by Dual Luciferase Reporter Assay System (Promega).

### In vivo subcutaneous tumor growth assay and IHC analysis

All animal studies were approved by the Institutional Animal Care and Use Committee of Sun Yat-sen University. 5-weeks-old BALB/c nude mice were purchased from Laboratory Animal Center of SYSU (Guangzhou, China) or Vital River (Beijing, China) and kept in SPF conditions. Control and u50535 overexpression PCDH plasmids were stably transfected into DLD1cells by lentivirus method. Then 4 × 10^6^ cells were subcutaneous injected to each BALB/c nude mice, respectively. The mice were sacrificed after 24 days and the xenograft tumors were presented and weighted. Each group contained seven mice. Tumor size was recorded every 3–4 days and calculated as volume = (length × width^2^)/2. Next, the tumor tissues were fixed, wrapped and cut into slices, followed by anti-Ki67 (CST) staining according to the routine IHC method. Ki67 positive cells numbers per 40× High Power Field (HPF) were calculated with Image J software, and five slices in each group were analyzed.

As for knockout xenograft animal assay, u50535 knockdown in DLD1 was constructed with lentiCRISPRv2 plasmid by CRISPR/cas9 method. Then 4 × 10^6^ cells were subcutaneous injected to each BALB/c nude mice. The mice were sacrificed after 35 days and the xenograft tumors were presented. Each group contained four mice. Then the following procedure was the same as above.

### In vivo lung metastasis assay

As for lung metastasis assays in vivo, 3 × 10^6^ DLD1 cells of u50535 overexpression/control and u50535 knockout/control were injected to tail vein of BALB/c nude mice. After 12 weeks, the mice were sacrificed and the lung tissues were fixed with neutral formalin and embedded with paraffin. Representative H&E images and metastasis tumor nodules on lung surface were presented. Based on the sizes and numbers of micro-metastasis, we scored each lung sections under microscope. We divided the tumor nodules into 4 grades based on size: Grade I <0.5 mm; Grade II 0.5–1.0 mm; Grade III 1.0–2.0 mm; Grade IV >2.0 mm. Thus, the score of lung micro metastasis = I × 1 + II × 2 + III × 3 + IV × 4. Each group contained five mice.

### RNA sequencing array and bioinformatics analysis

Whole transcriptome deep sequencing (RNAseq) was performed by KangChen Bio-tech Company (Shanghai, China) with Illumina Hiseq 4000. We designed the experiment with four groups, including silence pair DLD1siNC/siRNA and overexpression pair DLD1-pcDNA3.1/u50535OE. We analyzed the altered genes with Heat map analysis performed with the OmicShare tools, a free online platform for data analysis (http://www.omicshare.com/tools) . Then we also analyzed the data with Gene Ontology (GO) method in R software. GSEA results were performed with GSEA preranked tool analysis.

### Chromatin isolation by RNA purification (CHIRP)

CHIRP assay was performed as described before from Chang HY’s protocol^39^. Probes of antisense oligonucleotide were designed with 3′-BiotinTEG and divided into odd and even pools. Antisense oligonucleotides targeting LacZ RNA were used as negative control. Probes were showed in Supplementary Table S1. Cells (7 × 10^7^) were harvested and cross-linked with fresh 3% formaldehyde for 30 min and quenched with 1/10th volume of 1.25 M glycine for 5 min. The cells were lysed with 1 ml per 100 mg cell pellet of lysis buffer and sonicated by BIORUPOR sonicator for 60 cycles of 30 s. Cell lysis (1 ml/ 2 × 10^7^) was collected and added with 2 ml hybridization buffer and 100 pmol of each pool of probes for 5 h in 37 ° . RNA was pulled down by Dynabeads MyOne Streptavidin C1 for 30 min at 37 °C. And then beads were washed for 5 times. After last wash, 500 µl were used for RNA isolation and 500 µl for DNA isolation. RNA isolation were performed with proteinase-K-chloroform method. DNA isolation were performed with DNA extract kit (Qiagen). DNA was extracted and used for qPCR analysis with primers indicated in Supplementary Table S1.

### Statistical analysis

All statistical analysis was performed with SPSS 22.0 (Chicago, IL, USA) and GraphPad Prism 7.0 (California, USA). The significance of difference between groups was analyzed by two-tailed Student’s *t*-test, Chi-square test or Wilcoxon test, as appropriate. Overall survival was estimated by Kaplan–Meier analysis with log-rank test. Univariate and multivariate survival analysis were based on the Cox regression analyses model. Receiver operating characteristic (ROC) curve analysis was done by SPSS to set the cut-off value. *P* value <0.05 (two-sided) was considered to be significant.

## Electronic supplementary material

Supplementary-total

Supplementary-TableS1

Supplementary-TableS2

Supplementary-TableS3

Supplementary-TableS4
