# Supplementary material for: The novel long noncoding RNA u50535 promotes colorectal cancer growth and metastasis by regulating CCL20
Source: Cell Death Dis. 2018 Jul 3;9(7):751. doi: 10.1038/s41419-018-0771-y (PMC6030363; doi:10.1038/s41419-018-0771-y)
Supplement: Supplementary file 1 — Supplementary-total [file 41419_2018_771_MOESM1_ESM.docx]

**Supporting Information**

**The novel long noncoding RNA u50535 promotes colorectal cancer growth and metastasis by regulating CCL20**

Xihu Yu^1,2^, Zixu Yuan^3^, Zuli Yang^3^, Daici Chen^1,2^, Taewan Kim^6^, Yanmei Cui ^1,2^, Qianxin Luo^1,2^, Zhihang Liu^1,2^, Zihuan Yang^1,2^, Xinjuan Fan^4^, DianKe Chen^5,^*, Lei Wang^1,2,3,^*

**Supplementary Figures**

**S1**

**
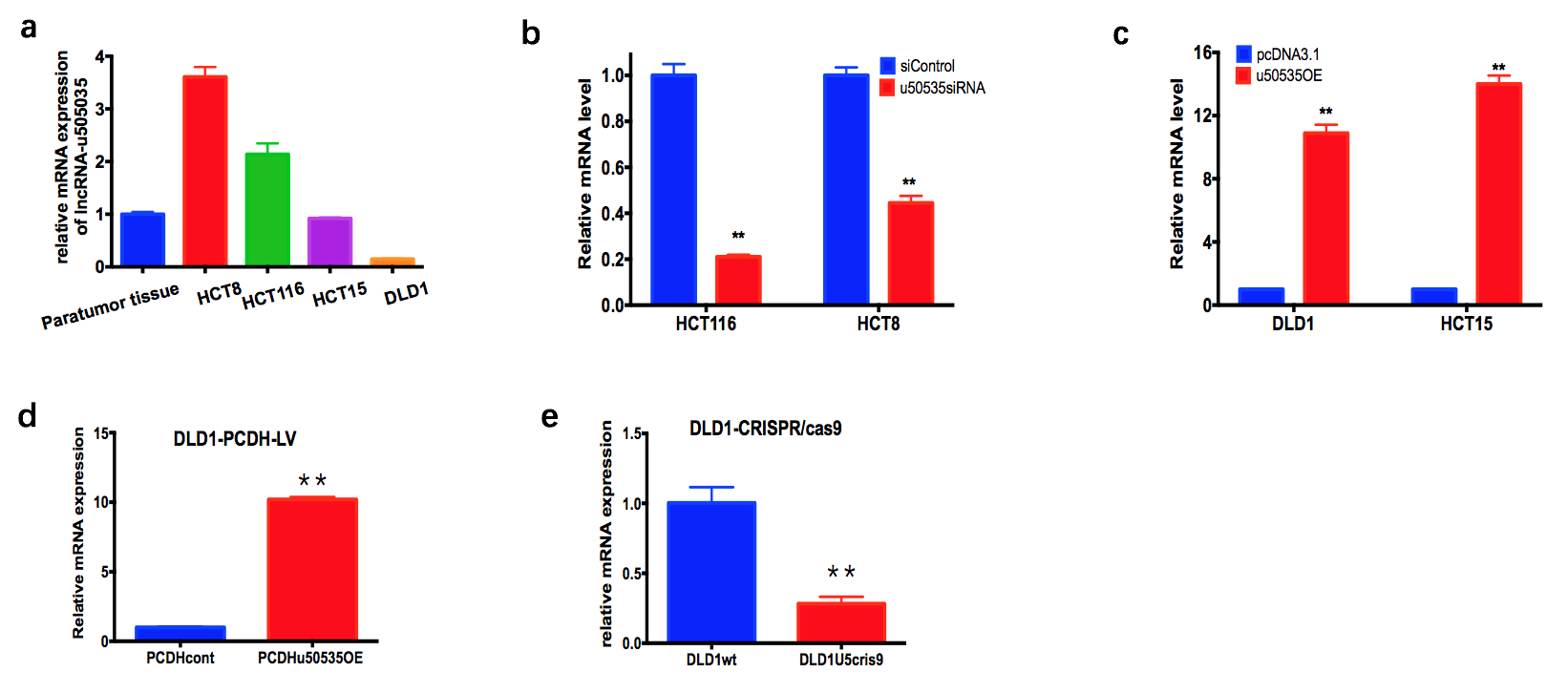
**

**Fig.S1.** **The LncRNA-u50535 expression levels in different CRC cells.** **a** LncRNA-u50535 expression was detected in four CRC cell lines and one paratumor tissue as relative control. Then we chose HCT116/ HCT8 as transient silence cell lines and HCT15/DLD1 as transient overexpression cell lines. **b** LncRNA-u50535 expression was significantly silenced by u50535siRNA transfection in HCT116 /HCT8. **c** LncRNA-u50535 was efficiently overexpressed by plasmid pcDNA3.1-u50535 transfection in DLD1/HCT15. **d** LncRNA-u50535 was stably overexpressed in DLD1 by lentivirus with PCDH-u50535. **e** LncRNA-u50535 was stably silenced in DLD1 by lentiCRISPRv2-u50535. Data is from three independent experiments and expressed as mean ± SD. “*”, P < 0.05; “**”, P < 0.01.

**S2**


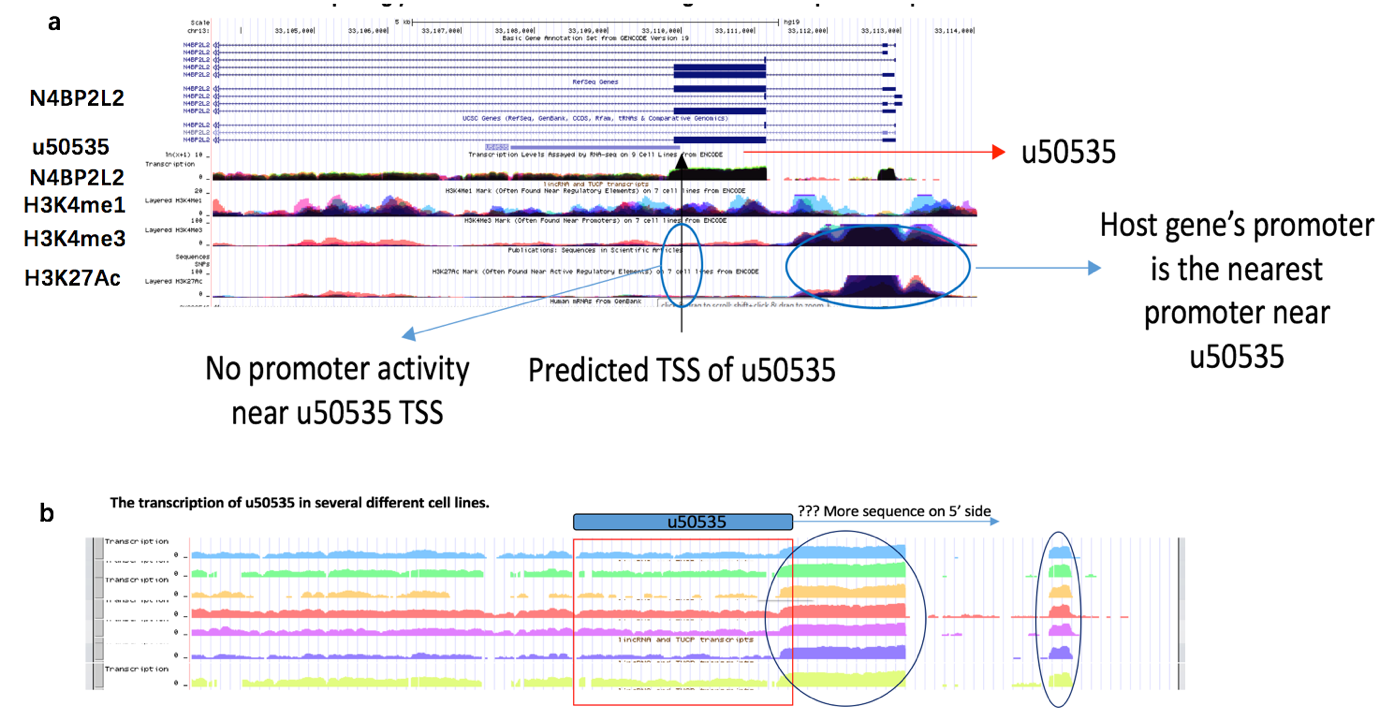


**Fig.S2.** **The detailed explanation of RACE results. a** Based on the UCSC database, the predicted size of u50535 is about 2.3kb, and it’s definitely an intronic lncRNA of host gene N4BP2L2. Based on the database of epigenetics at the bottom, the lncRNA does not show any independent and separate promoter activity near the transcription starting site (TSS) of u50535. It suggests that the transcription should rely on the promoter of its host gene. Thus, the lncRNA-u50535 might be produced during the alternative splicing of the host gene and the RACE product could be longer than its expected sequence. **b** It showed the transcription of u50535 in several different cell lines. In some cell lines, the expressed RNA is shorter than its expected sequence. On the other hand, no clear disruptions in RNA expression is shown in the predicted boundary of u50535 in some other cell lines. Therefore, we could expect that the lncRNA-u50535 could be longer in 5’side.

**S3**

**
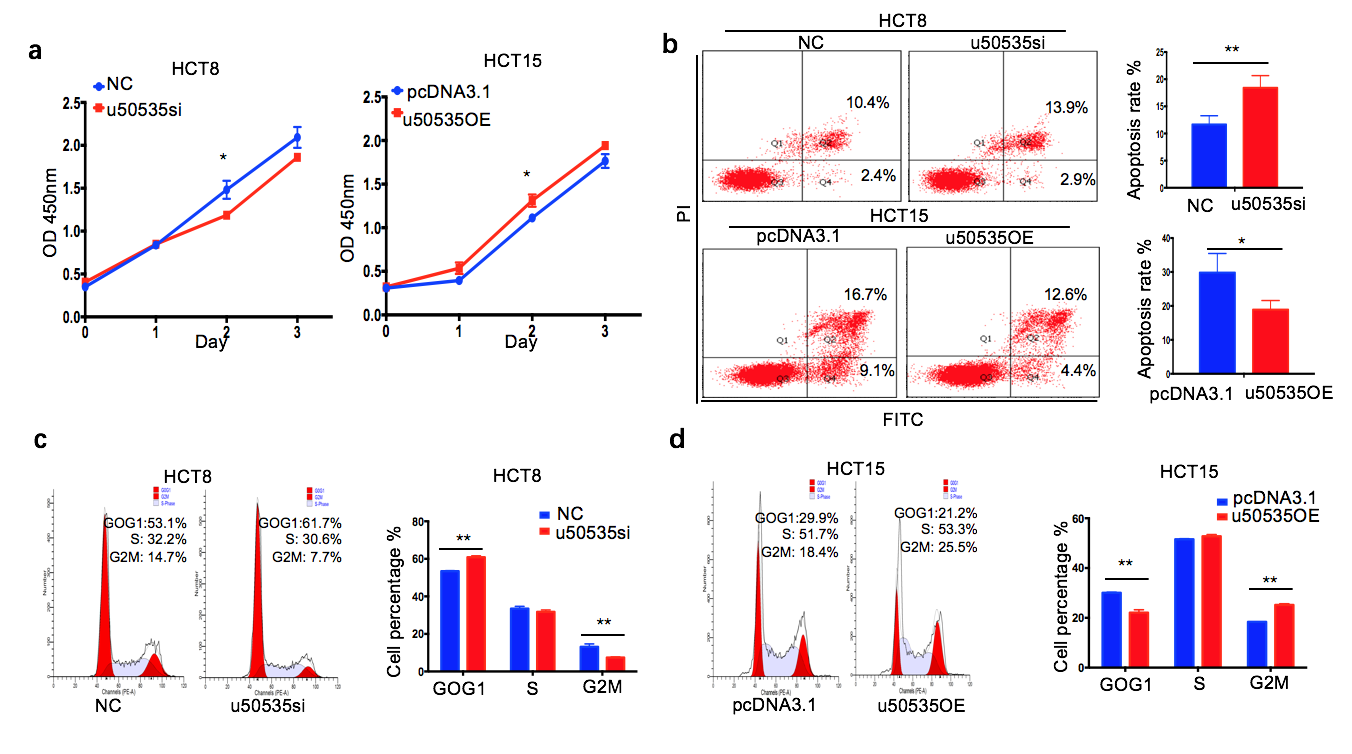
**

**Fig.S3. LncRNA-u50535 enhances CRC cell proliferation, inhibits apoptosis and accelerates in other cell lines. a** CCK8 assays revealed that u50535 silence inhibited HCT8 cell proliferation, while u50535 overexpression promoted HCT15 cell proliferation. **b** Apoptosis assays by flow cytometry indicated that u50535 silence increased apoptosis rate in HCT8, while u50535 overexpression decreased apoptosis rate in HCT15. **c, d** Cells in GOG1 period are mainly the non-proliferation cells. u50535 silence increased the percentage of HCT8 cells in GOG1 phase, whereas the u50535 overexpression inhibited the percentage of HCT15 cells in GOG1 phase. Data is from three independent experiments and expressed as mean ± SD. “*”, P < 0.05; “**”, P < 0.01.

**S4**


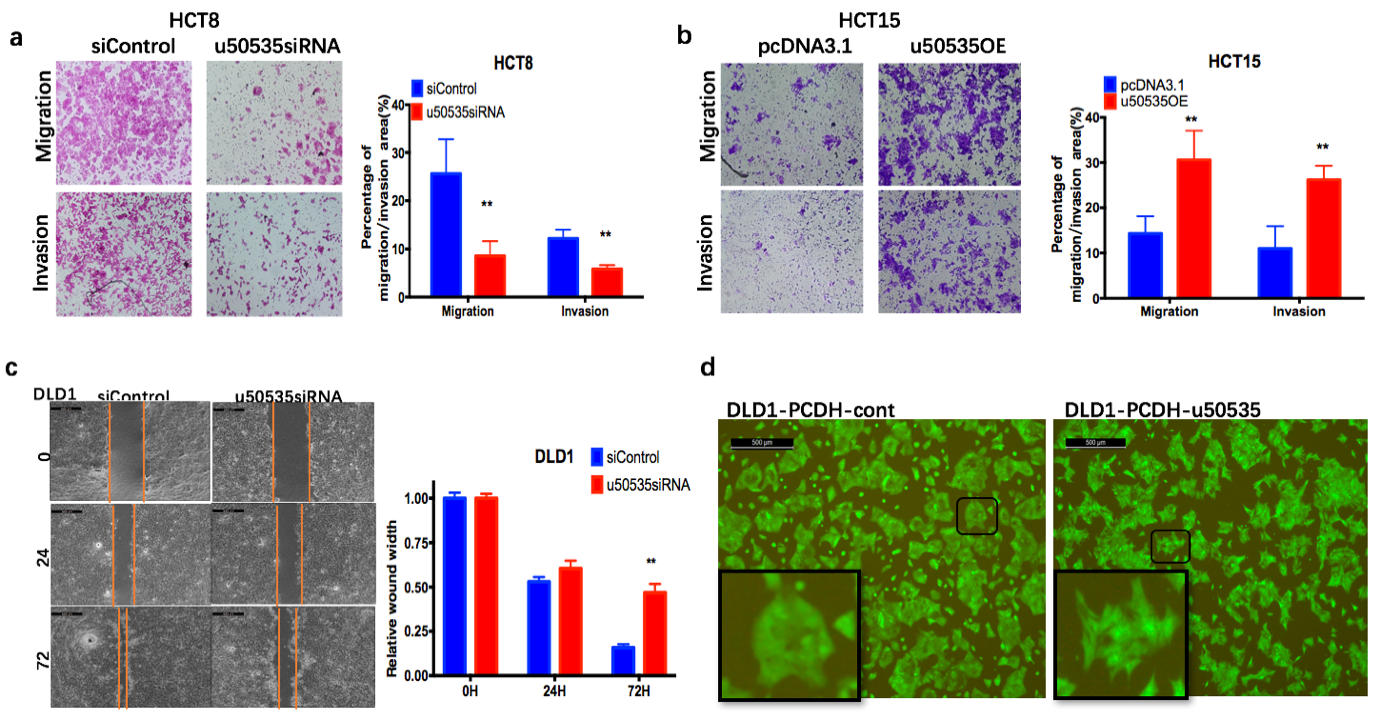


**
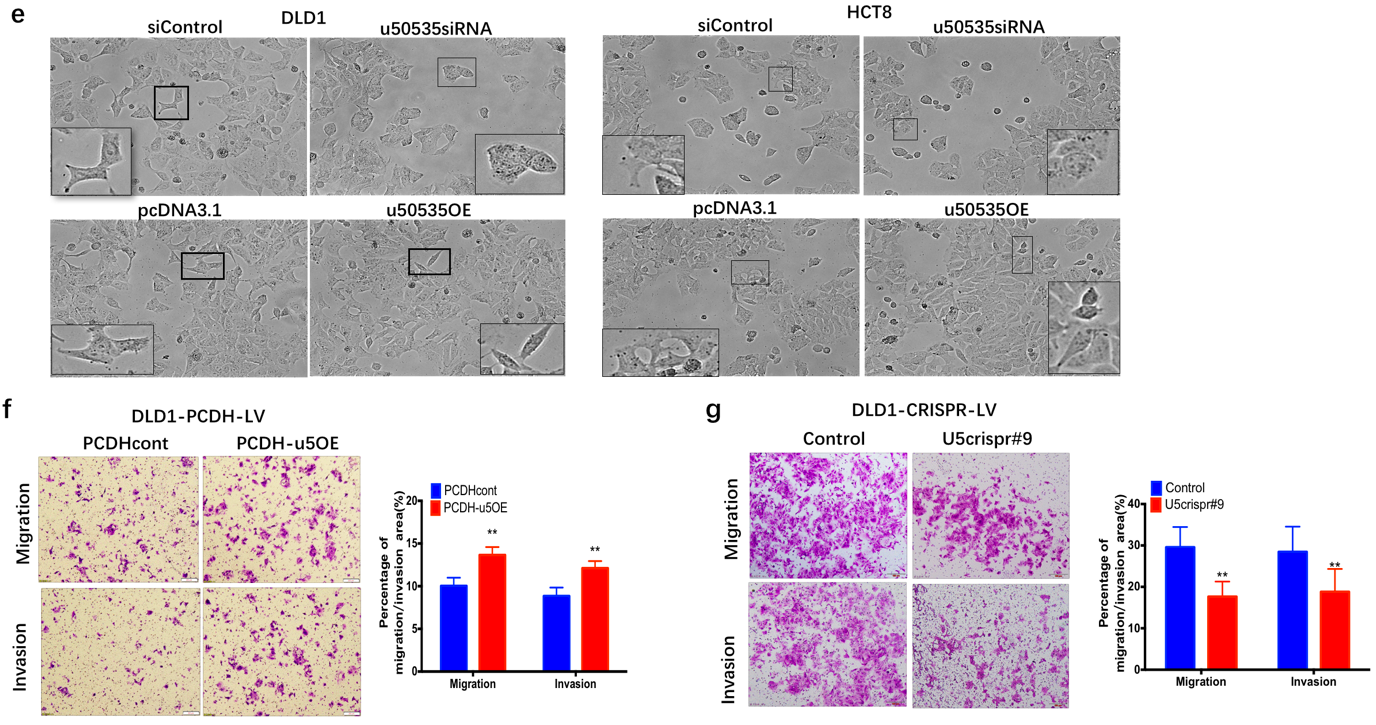
****Fig.S4. LncRNA-u50535 promotes CRC cell migration, invasion and morphology change in other cell lines.** **a, b** u50535 knockdown repressed both migration and invasion ability in HCT8, whereas an opposite result was found when u50535 expression was overexpressed in HCT15. **c** Wound healing test showed that u50535 knockdown would significantly slow down the wound healing ability in DLD1 in 72 hours. **d** Cell morphology of stable overexpression cell lines were captured at microscope. LncRNA-u50535 stably overexpression would result in a rough cell surface with some pseudopods, which would contribute to cell migration and invasion. **e** Cell morphology was captured at microscope after 24 hours transfection with u50535 siRNA or u50535 overexpression. LncRNA-u50535 silence would result in a smoother cell surface, more tightly packed cells, while u50535 overexpression would lead to an elongated and spindle-like cell surface, more free or loose cells. **f** Both migration and invasion cell numbers were increased after stably overexpression of u50535 by transwell assay. **g** Both migration and invasion cell numbers were decreased after stably silence of u50535. Data is from three independent experiments and expressed as mean ± SD. “*”, P < 0.05; “**”, P < 0.01.

**S5**


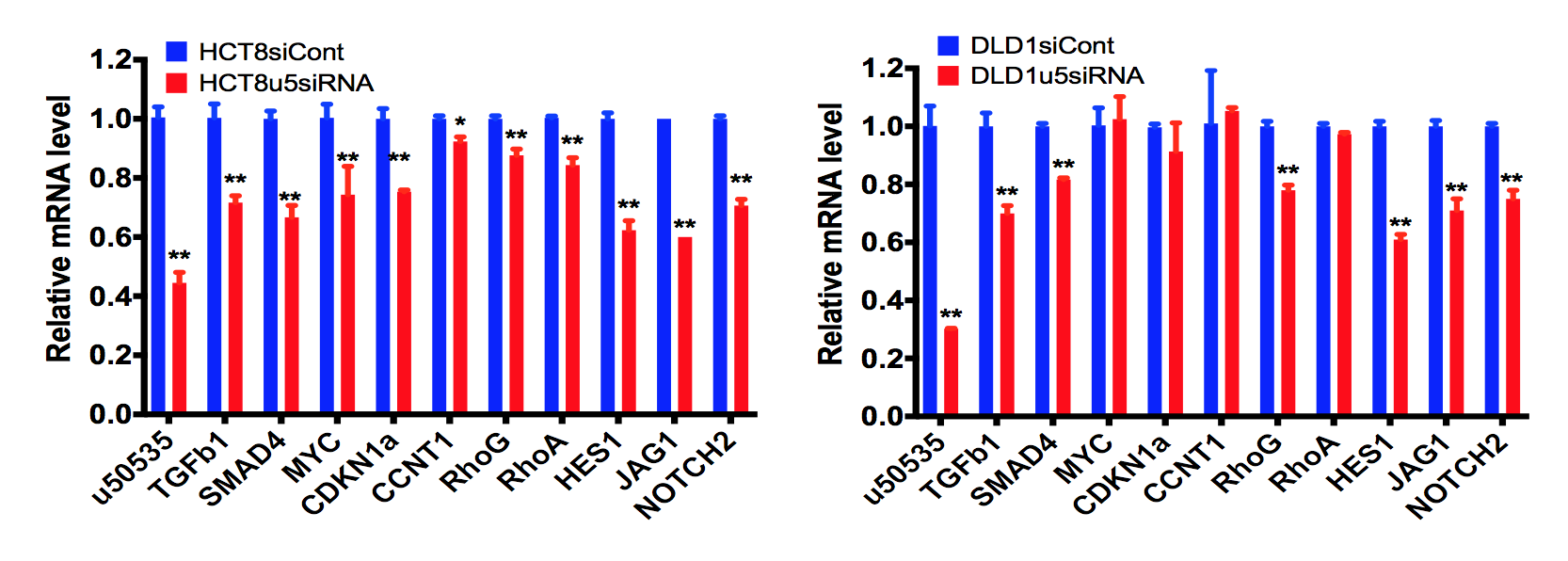


**Fig.S5.** The altered genes selected from GSEA were confirmed by real-time PCR in knockdown cell lines of u50535.

**S6**


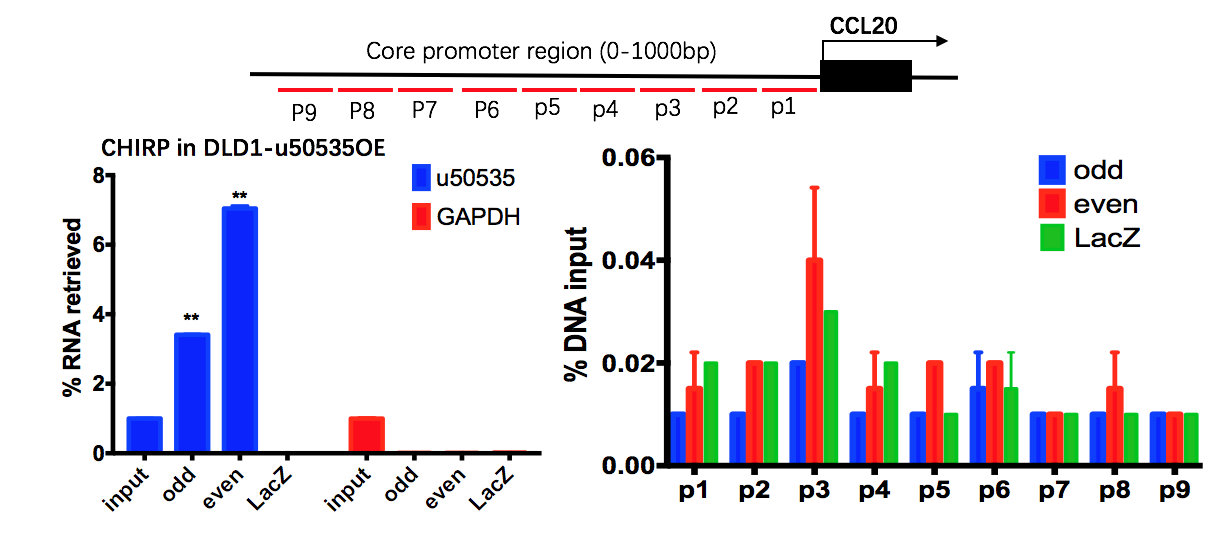


**Fig.S6.** CHIRP assay revealed that u50535 was significantly retrieved by u50535 antisense odd/even probes compared with negative control LacZ in u50535 overexpression cells(left), while chromatin/DNA of CCL20 core promoter region was not significantly enriched (right), indicating an indirect regulation.

**S7**

**
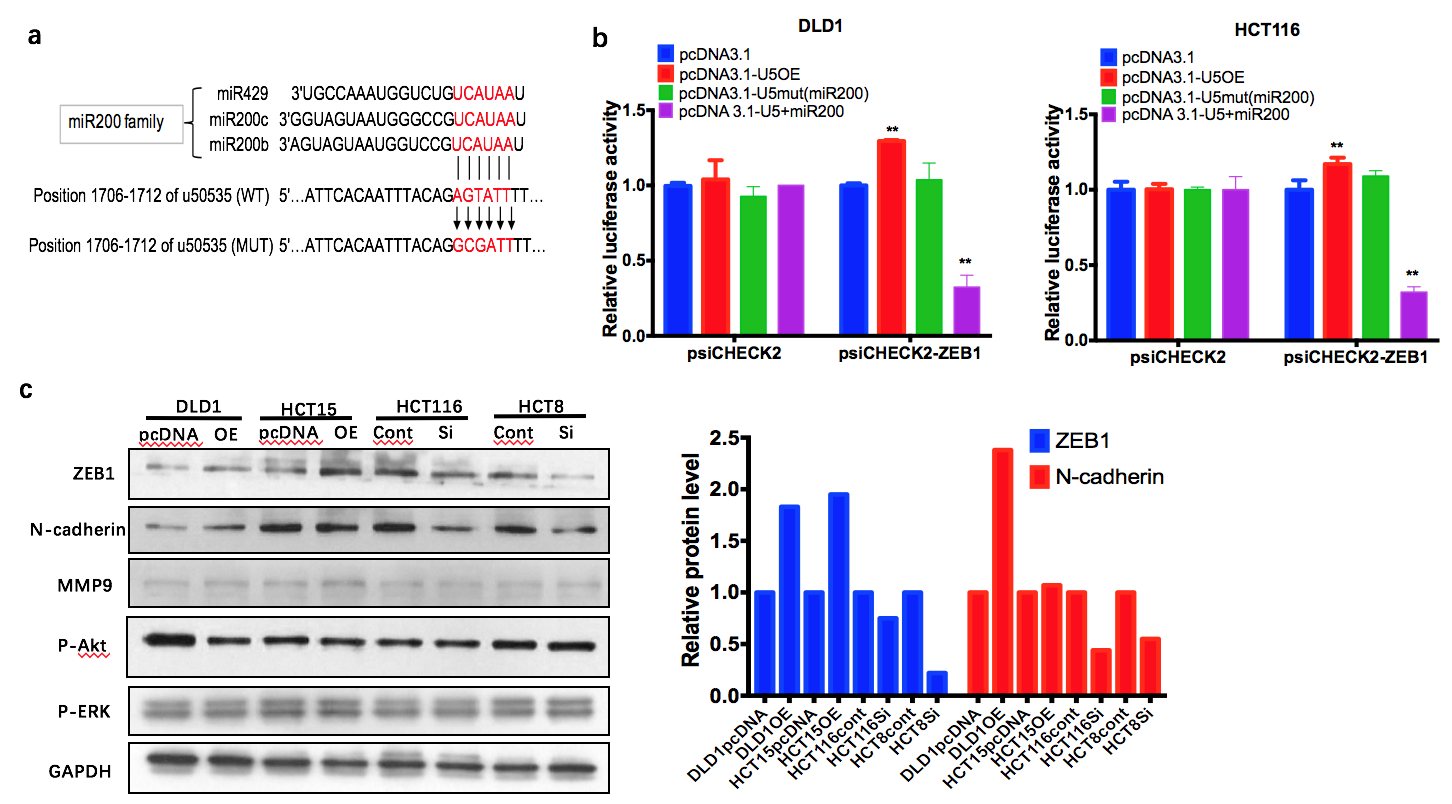
**

**Fig.S7. LncRNA-****u50535 in cytoplasm may serve as a sponge and promote ZEB1 expression by competing with miR200.** **a** Bio-information predicted the potential miRNA interacting with lncRNA-u50535. It showed that miR200 family (miR200b \miR429 \miR200c) may interact with region 1706-1712bp of u50535. In order to validating the interaction, we designed site mutation plasmid of u50535. **b** Dual luciferase analysis showed the interaction between u50535 and ZEB1 and miR200 in DLD1 and HCT116. The results indicated that u50535 can promote ZEB1 expression and miR200 can inhibit ZEB1. And at the same time, site mutation of u50535 will increase the free miR200 level, which will subsequently suppress the ZEB1 expression. **c** Western blotting showed the protein level of ZEB1, N-cadherin, MMP9, p-AKT, p-ERK in overexpression or silence cell lines. It indicated that u50535 can positive regulate ZEB1 and N-cadherin, thus promoting CRC cell migration and invasion.

**Supplementary Table S1 ： The list of primers and siRNAs.**

**Supplementary Table S2 ： RNAseq analysis mRNAs increased/reduced abundance (≥1.5-fold) in u50535 silence.**

**Supplementary Table S3 ：GO analysis of significant genes.**

**Supplementary Table S4 ：GSEA analysis of detail information.**
